# Inspiratory Efforts, Positive End-Expiratory Pressure, and External Resistances Influence Intraparenchymal Gas Redistribution in Mechanically Ventilated Injured Lungs

**DOI:** 10.3389/fphys.2020.618640

**Published:** 2021-02-09

**Authors:** Mariangela Pellegrini, Göran Hedenstierna, Anders Sune Larsson, Gaetano Perchiazzi

**Affiliations:** ^1^Hedenstierna Laboratory, Department of Surgical Sciences, Uppsala University, Uppsala, Sweden; ^2^Intensive Care Unit, Department of Anesthesia, Operation and Intensive Care, Uppsala University Hospital, Uppsala, Sweden; ^3^Hedenstierna Laboratory, Department of Medical Sciences, Uppsala University, Uppsala, Sweden

**Keywords:** assisted mechanical ventilation, lung heterogeneity, mild acute respiratory distress syndrome, CT imaging, self-induced lung injury

## Abstract

**Background:**

Potentially harmful lung overstretch can follow intraparenchymal gas redistribution during mechanical ventilation. We hypothesized that inspiratory efforts characterizing spontaneous breathing, positive end-expiratory pressure (PEEP), and high inspiratory resistances influence inspiratory intraparenchymal gas redistribution.

**Methods:**

This was an experimental study conducted on a swine model of mild acute respiratory distress syndrome. Dynamic computed tomography and respiratory mechanics were simultaneously acquired at different PEEP levels and external resistances, during both spontaneous breathing and controlled mechanical ventilation. Images were collected at two cranial–caudal levels. Delta-volume images (ΔVOLs) were obtained subtracting pairs of consecutive inspiratory images. The first three ΔVOLs, acquired for each analyzed breath, were used for the analysis of inspiratory pendelluft defined as intraparenchymal gas redistribution before the start of inspiratory flow at the airway opening. The following ΔVOLs were used for the analysis of gas redistribution during ongoing inspiratory flow at the airway opening.

**Results:**

During the first flow-independent phase of inspiration, the pendelluft of gas was observed only during spontaneous breathing and along the cranial-to-caudal and nondependent-to-dependent directions. The pendelluft was reduced by high PEEP (*p* < 0.04 comparing PEEP 15 and PEEP 0 cm H_2_O) and low external resistances (*p* < 0.04 comparing high and low external resistance). During the flow-dependent phase of inspiration, two patterns were identified: (1) *gas displacing* characterized by large gas redistribution areas; (2) *gas scattering* characterized by small, numerous areas of gas redistribution. *Gas displacing* was observed at low PEEP, high external resistances, and it characterized controlled mechanical ventilation (*p* < 0.01, comparing high and low PEEP during controlled mechanical ventilation).

**Conclusions:**

Low PEEP and high external resistances favored inspiratory pendelluft. During the flow-dependent phase of the inspiration, controlled mechanical ventilation and low PEEP and high external resistances favored larger phenomena of intraparenchymal gas redistribution (gas displacing) endangering lung stability.

## Introduction

The intraparenchymal gas redistribution occurring in absence of flow at the airway opening has been defined for the first time in 1956 and named pendelluft (Otis et al., 1956). The magnitude of this phenomenon is closely related to the heterogeneity of lung injury and to the ventilatory settings. The heterogeneity in regional compliance and resistance, characterizing injured lungs (Harada et al., 1983; Sala et al., 2002; Perlman et al., 2010; Vyshedskiy and Murphy, 2012; Yoshida et al., 2016b), leads to unstable acinar structures and transient pressure gradients among adjacent lung regions, consequently causing gas redistribution within the lung parenchyma (Yoshida et al., 2016a; Brochard et al., 2017). Moreover, suboptimal ventilatory settings and strong inspiratory efforts further enhance heterogeneity between contiguous regions and promote lung instability (Otis et al., 1956; Mead et al., 1970; Ultman et al., 1988; Greenblatt et al., 2014). Recent studies show harmful inspiratory pendelluft following high inspiratory efforts when spontaneous breathing is allowed in mechanically ventilated subjects with acute respiratory distress syndrome (ARDS) (Yoshida et al., 2013; Morais et al., 2018). Consequently, the scientific community debates whether and to which extent to allow spontaneous breathing in mechanically ventilated subjects affected by ARDS (Papazian et al., 2010; Haren et al., 2019; Moss et al., 2019; Schepens et al., 2019).

To investigate all possible factors causing intraparenchymal gas redistribution is of crucial importance for understanding the mechanisms leading to ventilator-induced lung injury. In the current experimental study, we investigated if spontaneous inspiratory efforts, positive end-expiratory pressure (PEEP), and external resistances influenced onset and magnitude of intraparenchymal gas redistribution:

(a) during the first isovolumetric, flow-independent phase of the inspiration defined as pendelluft (Yoshida et al., 2013);
(b) during ongoing inspiratory flow at the airway opening.

## Materials and Methods

The current study was approved by the regional animal ethics committee (No. C 46_14) and performed at the Hedenstierna Laboratory (Uppsala University, Sweden). Detailed information about the methods has been provided in the online data supplement.

### Experimental Setting and Protocol

Six anesthetized, mechanically ventilated pigs (29.9 ± 2.6 kg) underwent repeated lung lavages (30 ml/kg of warmed isotonic saline at 37°C) to create a surfactant-deficient mild ARDS injury (PO_2_/F_I_O_2_ of 250 mmHg at a PEEP of 5 cm H_2_O). The animals were placed in supine position. Spontaneous breathing (continuous positive airway pressure) and controlled mechanical ventilation in fully paralyzed conditions (pressure-controlled ventilation) were applied at comparable tidal volumes and respiratory rates. Spontaneous breathing was performed at two different external airway resistances: high (SB-HighR) and low (SB-LowR) resistance. SB-LowR was achieved by applying an endotracheal tube with an internal diameter of 9 mm. SB-HighR was achieved by applying an endotracheal tube with an internal diameter of 6 mm. During both spontaneous breathing and controlled mechanical ventilation, six PEEP levels (15, 12, 9, 6, 3, and 0 cm H_2_O) were applied. A steady state was always restored between two consecutive tested ventilatory conditions. Respiratory mechanics, electrical activity of the diaphragm (EAdi), and dynamic CT scans were simultaneously recorded. Dynamic CT images were recorded 1 (L1) and 4 cm (L4) above the diaphragmatic dome, with an acquisition rate of 20 images per second.

### Data Analysis

The volume tracings derived from simultaneously acquired CT scans allowed the synchronization between CT images and spirometric data, as done in Pellegrini et al. (2017), thus allowing breath-by-breath analysis of tracings and images in-phase. All acquired inspiratory CT images were further processed to obtain delta-volume images (ΔVOLs) between consecutive CT images. Each delta volume was characterized by a 0.05-s time frame.

### Intraparenchymal Gas Redistribution Before the Onset of Inspiratory Flow (Pendelluft)

**FIGURE 1:**
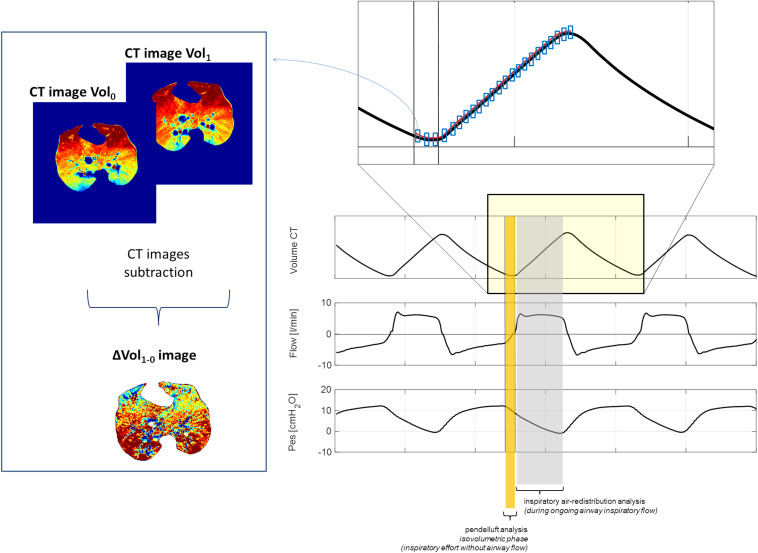
Methods. Left panel: Methods to obtain delta-volume images. Right panel: Isovolumetric flow-independent phase defined as pendelluft (yellow rectangle) of the inspiration and the flow-dependent phase of the inspiration (gray rectangle).

The inspiratory pendelluft was defined as intraparenchymal gas redistribution when the inspiratory effort has not yet induced an inspiratory flow at the airway opening (Otis et al., 1956; Yoshida et al., 2013). During this phase, the internal redistribution of gas is possible. The time related to auto-PEEP was calculated to estimate the time of isovolumetric inspiration (see Figure 1). During spontaneous breathing, dynamic auto-PEEP was calculated as the difference in Pes between the point of zero flow at end expiration and the onset of the subsequent inspiratory flow. During controlled mechanical ventilation, auto-PEEP was estimated by calculating the difference in airway pressure in the time frame between end expiration and the onset of inspiratory flow (Rossi et al., 1995). The number of CT images at the beginning of the inspiration to be included in the pendelluft analysis was determined based on the longest recorded time necessary to overcome auto-PEEP in the entire group of studied animals. To explore the inspiratory pendelluft, four CT images (and the corresponding three ΔVOLs, covering the first 0.15 s of each inspiration) were selected at the beginning of each inspiration (see Figure 1). Each ΔVOL was then divided into four equally sized regions of interest (ROI1, ROI2, ROI3, and ROI4), with ROI1 being the most nondependent and ROI4 the most dependent one. For each ROI, gas flow was computed and normalized to the extension of the corresponding ROI: (ml of gas/0.05 s/mm^3^ of ROI extension). Combining four gravitational ROIs and two cranial–caudal levels, a three-dimensional assessment of intraparenchymal gas redistribution was possible.

### Intraparenchymal Gas Redistribution During Ongoing Inspiratory Flow

To reveal patterns of gas redistribution during ongoing inspiratory flow at the airway opening, the quadtree decomposition algorithm was applied to the ΔVOLs (see Figure 1). The quadtree decomposition is a standard algorithm in image processing (18, 19) that iteratively divides the image into four equal squares until a predetermined criterion of homogeneity is satisfied. If the criterion is not satisfied, the analyzed image portion is further divided into four squares. The criterion of homogeneity was defined as a difference in flow between contiguous areas equal to or lower than 10% (corresponding to a flow difference equal to or lower than 2 × 10^–5^ ml/0.05 s). The mean area of all squares (AreaSq) obtained by the quadtree decomposition algorithm and expressed in cm^2^ was used to define two patterns of intraparenchymal gas redistribution. For mean AreaSq equal to or lower than the arbitrary threshold of 2 cm^2^, the gas redistribution pattern was defined as *gas scattering*; for mean AreaSq higher than 2 cm^2^, the pattern of gas redistribution was defined as *gas displacing*.

### Statistics

Normal distribution was confirmed by the one-sample Kolmogorov–Smirnov test (α = 0.05). The analysis of variance was used to test statistical differences. Bonferroni’s correction for multiple comparisons was applied when needed. Descriptive statistics were reported using mean (± SEM).

## Results

There were no differences in transpulmonary pressure between spontaneous breathing and controlled mechanical ventilation (see Table 1). This finding made the two tested ventilatory modes comparable in the following phases of the study.

**TABLE 1:** Respiratory mechanics for each studied ventilatory modalities and PEEP level: (A) spontaneous breathing at low external airway resistance; (B) spontaneous breathing at high external airway resistance; (C) controlled mechanical ventilation.

Spontaneous breathing low resistance							

PEEP	Tidal volume (mL)	Unfiltered EAdi peak (uV)	Neuroventilatory efficiency (ml/uV)	ΔPes (cm H_2_O)	Auto-PEEP (cm H_2_O)	Time of no-flow (s)	Ptp (cm H_2_O)
15	225 ± 7	17.2 ± 1.8^†^	13.1 ± 1.5	−5.06 ± 0.48*	0.01 ± 0.01	0.00 ± 0.01	20.06 ± 0.48^†^
12	220 ± 7	13.8 ± 1.9	16.0 ± 1.4*	−5.21 ± 0.27*	0.25 ± 0.14	0.02 ± 0.01	17.21 ± 0.27^†^
9	219 ± 8	14.1 ± 1.8	15.6 ± 1.4*	−6.94 ± 0.89	0.25 ± 0.12	0.02 ± 0.01	15.94 ± 0.89^†^
6	211 ± 9	13.5 ± 1.6	15.6 ± 1.4*	−7.48 ± 0.83	0.56 ± 0.19*	0.03 ± 0.01	13.48 ± 0.83^†^
3	180 ± 9*	14.5 ± 1.0	12.4 ± 1.0	−8.29 ± 0.75	1.02 ± 0.17*	0.06 ± 0.01*	11.29 ± 0.75^†^
0	167 ± 9*	14.0 ± 0.7	12.0 ± 0.9	−7.91 ± 0.97	1.62 ± 0.57*	0.09 ± 0.01*	7.91 ± 0.97^†^

**Spontaneous breathing high resistance**							

**PEEP**	**Tidal volume (ml)**	**Unfiltered EAdi peak (uV)**	**Neuroventilatory efficiency (ml/uV)**	**ΔPes (cm H_2_O)**	**Auto-PEEP (cm H_2_O)**	**Time of no-flow (s)**	**Ptp (cm H_2_O)**

15	214 ± 4	16.6 ± 1.9	12.9 ± 1.1*	−6.98 ± 1.29^†^	0.47 ± 0.12	0.04 ± 0.01	21.98 ± 0.48*
12	215 ± 9	15.8 ± 2.1	13.6 ± 0.9*	−8.28 ± 1.88	0.84 ± 0.12	0.05 ± 0.01	20.28 ± 0.27*
9	215 ± 5	15.4 ± 1.6	13.9 ± 0.9*	−8.93 ± 1.68	0.81 ± 0.14	0.06 ± 0.01	17.93 ± 0.89^†^
6	207 ± 9	14.3 ± 1.5	14.5 ± 1.3*	−8.74 ± 1.02	1.48 ± 0.28*	0.06 ± 0.01	14.74 ± 0.83
3	197 ± 7	16.6 ± 1.3	11.8 ± 1.0	−10.59 ± 1.94	1.93 ± 0.36*	0.10 ± 0.01	13.59 ± 0.75
0	172 ± 4^†^	17.8 ± 0.6*	9.7 ± 0.9^†^	−11.20 ± 2.50	2.02 ± 0.29*	0.13 ± 0.01	11.20 ± 0.97^†^

**Mechanical ventilation**							

**PEEP**	**Tidal volume (ml)**	**EAdi peak (uV)**	**ΔPes (cm H_2_O)**	**Auto-PEEP (cm H_2_O)**	**Time of no-flow (s)**	**Pplat (cm H_2_O)**	**Ptp (cm H_2_O)**

15	250	0.6 ± 0.1	0.89 ± 0.42	0.10 ± 0.02	0.01 ± 0.01	21.42 ± 0.89^†^	20.53 ± 0.78^†^
12	250	0.6 ± 0.0	0.95 ± 0.39	0.17 ± 0.03	0.02 ± 0.00	18.22 ± 0.94^†^	17.27 ± 0.85^†^
9	250	0.7 ± 0.0	1.02 ± 0.50	0.17 ± 0.03	0.02 ± 0.00	15.01 ± 1.00^†^	13.99 ± 0.86^†^
6	200	0.6 ± 0.0	0.74 ± 0.37	0.22 ± 0.03	0.03 ± 0.00	12.31 ± 1.01^†^	11.57 ± 0.94^†^
3	200	0.7 ± 0.1	0.71 ± 0.29	0.22 ± 0.03	0.03 ± 0.00	9.32 ± 0.97^†^	8.61 ± 0.93^†^
0	200	0.6 ± 0.1	0.74 ± 0.28	0.36 ± 0.03*	0.03 ± 0.00	6.48 ± 0.97^†^	5.74 ± 0.93^†^

Data collected from all six animals (mean ± SEM). The neuroventilatory efficiency is calculated as tidal volume/unfiltered EAdi peak. ANOVA (α = 0.05) with Bonferroni’s correction for multiple comparisons. PEEP, positive end-expiratory pressure; EAdi, electrical activity of the diaphragm. *Groups of not statistically different values, statistically different from others values of the same variable. ^†^Values that are statistically different compared to other values of the same variable.

### Intraparenchymal Gas Redistribution Before the Onset of Inspiratory Flow (Pendelluft)

**FIGURE 2:**
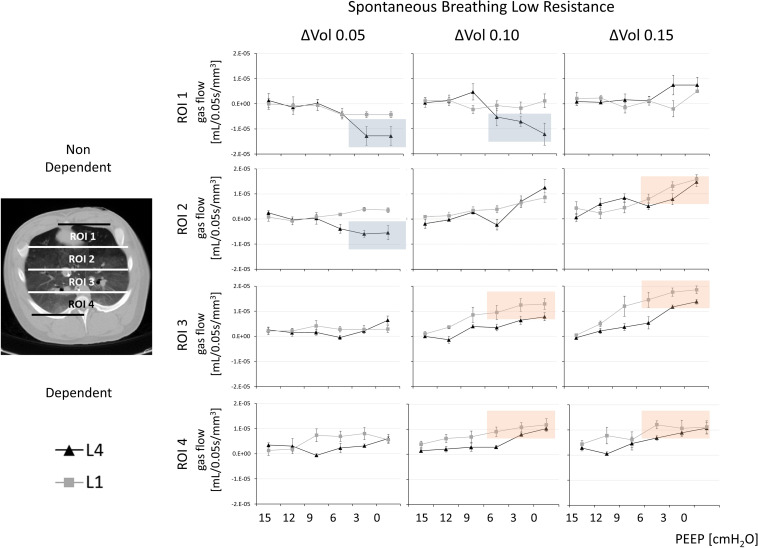
Gas redistribution during the isovolumetric phase of inspiration: the pendelluft analysis. Pendelluft during spontaneous breathing and low external airway resistance. Based on the estimated time to the onset of the inspiratory flow at the beginning of the inspiration, three delta volumes at, respectively, 0.05, 0.10, and 0.15 s (ΔVOL_0.05_, ΔVOL_0.10_, and ΔVOL_0.15_) were analyzed. Each delta-volume image was divided into four regions of interest (ROIs) along the gravitational axis (ROI1, ROI2, ROI3, and ROI4). The total amount of gas flow normalized for the corresponding ROI volume (ml/mm^3^; mean ± SE) (y-axis) was calculated at six different positive end-expiratory pressure (PEEP) levels (x-axis). The gray line indicates L1: 1 cm from the diaphragmatic dome. The black line indicates L4: 4 cm from the diaphragmatic dome. Complementary information in Figure 3 and Supplementary Figure 1.

**FIGURE 3:**
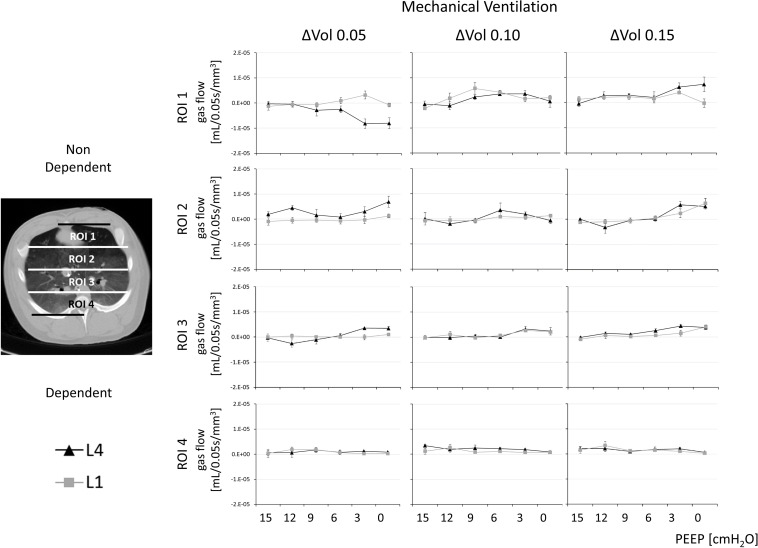
Gas redistribution during the isovolumetric phase of inspiration: the pendelluft analysis. Pendelluft during controlled mechanical ventilation in paralyzed conditions. Complementary information in Figure 2 and Supplementary Figure 1.

During controlled mechanical ventilation, auto-PEEP was always very low (0.1–0.4 cm H_2_O), independently from the PEEP level. During spontaneous breathing, the longest estimated no-flow time at the airway opening at the beginning of inspiration, corresponding to the highest recorded auto-PEEP (2 cm H_2_O), was 0.13 s (see Tables 1, 2 and Supplementary Tables 1–3). Consequently, considering a time frame of 0.05 s for each ΔVOL, the first three ΔVOLs (at 0.05, 0.10, and 0.15 s, respectively, named ΔVOL_0.05_, ΔVOL_0.10_, and ΔVOL_0.15_) of each analyzed inspiration were selected for the analysis of pendelluft (see Figures 1–3).

**TABLE 2:** Auto-PEEP and time of no-flow at the beginning of the inspiration.

	Spontaneous breathing low resistance		Spontaneous breathing high resistance		Mechanical ventilation	
			
	Auto-PEEP (cm H_2_O)	Time of no-flow (s)	Auto-PEEP (cm H_2_O)	Time of no-flow (s)	Auto-PEEP (cm H_2_O)	Time of no-flow (s)

PEEP	Mean ± SEM	Mean ± SEM	Mean ± SEM	Mean ± SEM	Mean ± SEM	Mean ± SEM
15	0.01 ± 0.01	0.00 ± 0.01	0.47 ± 0.12	0.04 ± 0.01	0.10 ± 0.02	0.01 ± 0.005
12	0.25 ± 0.14	0.02 ± 0.01	0.84 ± 0.12	0.05 ± 0.01	0.17 ± 0.03	0.02 ± 0.004
9	0.25 ± 0.12	0.02 ± 0.01	0.81 ± 0.14	0.06 ± 0.01	0.17 ± 0.03	0.02 ± 0.003
6	0.56 ± 0.19*	0.03 ± 0.01	1.48 ± 0.28*	0.06 ± 0.01	0.22 ± 0.03	0.03 ± 0.004
3	1.02 ± 0.17*	0.06 ± 0.01*	1.93 ± 0.36*	0.10 ± 0.01*	0.22 ± 0.03	0.03 ± 0.002
0	1.62 ± 0.57*	0.09 ± 0.01*	2.02 ± 0.29*	0.13 ± 0.01*	0.36 ± 0.03*	0.03 ± 0.003

Data collected from all six animals (mean ± SEM). ANOVA (α = 0.05) with Bonferroni’s correction for multiple comparisons. Auto-PEEP, intrinsic positive end-expiratory pressure; MV, controlled mechanical ventilation. *Statistical differences.

During spontaneous breathing, for PEEP levels equal to or higher than 9 cm H_2_O, all ROIs were characterized by a minimal, nonsignificant change in regional volumes (with a regional gas flow < 1.42E–06 ml/mm^3^ and nonstatistical differences between PEEP 15 and PEEP 9 cm H_2_O in ROI1 and ROI2, ΔVOL_0.05_ at SB-LowR) (see Figure 2 and Supplementary Figure 1). This was true for both SB-LowR and SB-HighR (with a regional gas flow < 1.29E−06 ml/mm^3^ and nonstatistical differences between PEEP 15 and PEEP 9 cm H_2_O in ROI1 and ROI2, ΔVOL_0.05_ at SB-HighR).

During spontaneous breathing at PEEP levels lower than 9 cm H_2_O, a nondependent cranial (ROI1 and ROI2 at L4) deflation (during ΔVOL_0.05_ and ΔVOL_0.10_) was quasi-simultaneous with a dependent caudal (ROI3 and ROI4 at L1) inflation (during ΔVOL_0.10_ and ΔVOL_0.15_) (see Figure 2 and Supplementary Figure 1, blue squares to highlight deflation, red squares to highlight inflation and Supplementary Tables 1, 2). This intraparenchymal gas redistribution was significantly higher during SB-HighR than during SB-LowR (*p* < 0.03 for statistical differences between gas flow during SB-LowR and SB-HighR and PEEP equal or lower than 6 cm H_2_O for both ΔVOL_0.05_ ROI1 and ΔVOL_0.15_ ROI4; see Supplementary Table 3). During controlled mechanical ventilation, independently from the selected PEEP level, no pendelluft was observed (see Figure 3).

### Intraparenchymal Gas Redistribution During Ongoing Inspiratory Flow

**FIGURE 4:**
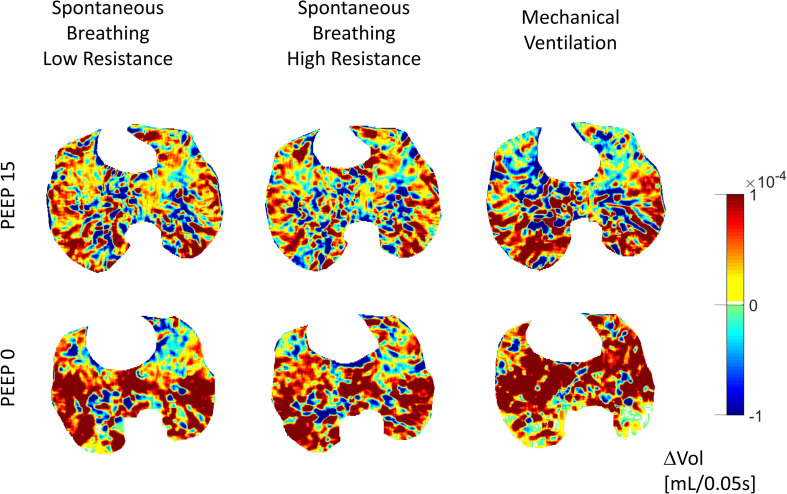
Gas redistribution during ongoing inspiratory flow: delta-volume images (ΔVOL). Large and few areas of gas redistribution (gas displacing) characterized low PEEP, high external resistances (SB-HighR), and controlled mechanical ventilation. Small and diffused areas of gas redistribution (gas displacing) characterized high PEEP, low external resistances (SB-LowR), and spontaneous breathing. Color scale: inflating areas in yellow–orange–red and deflating areas in green–sky–blue.

Sequences of ΔVOLs allowed a qualitative analysis of the intraparenchymal gas redistribution throughout inspiration. A visual inspection of sequences of the ΔVOLs showed continuous gas redistribution throughout the entire inspiration; multiple temporary and local transients of gas redistribution continuously occurred independently from the ventilatory conditions (see Figure 4).

The quadtree decomposition algorithm identified two gas-redistribution patterns during ongoing inspiratory flow at the airway opening: (1) few and large areas of gas redistribution, referred to as *gas displacing* (AreaSq equal to or larger than 2 cm^2^); (2) several and small areas of gas redistribution, referred to as *gas scattering* (AreaSq smaller than 2 cm^2^).

**FIGURE 5:**
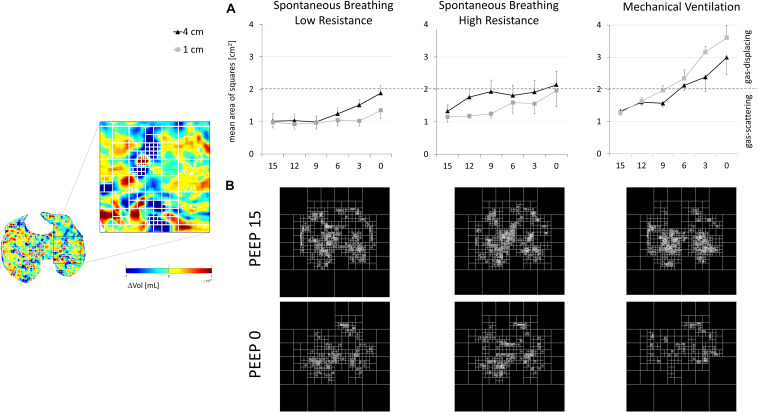
Flow-dependent gas redistribution: quadtree decomposition. Left panel: The quadtree decomposition algorithm applied to delta-volume images (ΔVOL). Right panel: **(A)** AreaSq equal to 2 cm^2^ (dashed gray line) was defined as the limit to distinguish between the two patterns of gas redistribution: gas scattering (mean AreaSq < 2 cm^2^) and gas displacing (mean AreaSq > 2 cm^2^). Gas scattering mainly characterized *spontaneous breathing low resistance* (left panel), both at high PEEP levels (vs low PEEP levels) and at 1 cm (vs 4 cm) from the diaphragm. During *spontaneous breathing high resistance* (central panel), gas scattering became closer to the limit of 2 cm^2^. Gas displacing mainly characterized low PEEP during *controlled mechanical ventilation* (right panel). This was true mainly in regions closer to the diaphragm. **(B)** Quadtree decomposition of a representative animal during the three studied ventilatory modalities. The figure compares PEEP 15 (the highest studied PEEP—upper panel) and PEEP 0 (the lowest studied PEEP—lower panel). The number of iterative divisions of squares was higher for PEEP 15 than for PEEP 0.

During spontaneous breathing and low external resistances (SB-LowR), high PEEP levels (equal to or higher than 6 cm H_2_O for L4 and equal to or higher than 3 cm H_2_O for L1) were associated with small value of AreaSq (gas scattering) (see Figure 5 and Supplementary Table 4). A higher external resistance (SB-HighR) applied to the airway caused a significant increase in AreaSq (gas displacing) for all PEEP levels equal to or lower than 12 cm H_2_O (see Supplementary Table 5). This was seen at both one (L1) and four (L4) cm cranially to the diaphragmatic dome.

Controlled mechanical ventilation caused a considerable increase in AreaSq (gas displacing) (see Figure 5 and see Supplementary Table 5). At PEEP of 15 cm H_2_O, the AreaSq value during controlled mechanical ventilation was not significantly different from that during spontaneous breathing. For lower PEEP levels, the AreaSq value was significantly larger (toward the gas-displacing limit) during controlled mechanical ventilation than during spontaneous breathing, independently from the distance from the diaphragm (L1 and L4) (see Figure 5 and see Supplementary Table 6).

The changes toward gas displacing characterizing low PEEP, already described during spontaneous breathing, were also maintained during controlled mechanical ventilation. However, gas displacing was much more emphasized during controlled mechanical ventilation than during spontaneous breathing (see Figure 5 and Supplementary Tables 4, 5).

During spontaneous breathing, the lung regions close to the diaphragm (L1) showed more gas scattering (small AreaSq) than the cranial regions (L4). Conversely, controlled mechanical ventilation caused larger gas displacing (large AreaSq) in caudal regions proximal to the diaphragm (L1) than in more cranial ones (see Figure 5 and Supplementary Table 6).

## Discussion

In the current study, we analyzed temporal and spatial patterns of intraparenchymal gas redistribution in an animal model of mild ARDS. We showed that ventilation of heterogeneous lungs caused transient micromechanical events that trigger the onset of multifold, temporary intraparenchymal gas-redistribution phenomena. This continuously occurred throughout the whole inspiration, during both spontaneous breathing and controlled mechanical ventilation. Therefore, the highly debated pendelluft (Yoshida et al., 2013, 2016a) was only one of the possible inspiratory gas-redistribution phenomena observed. The intraparenchymal gas redistribution occurring during inspiratory flow exhibited two different patterns: (1) gas displacing (i.e., few large areas of gas redistribution) or (2) gas scattering (i.e., several small areas of gas redistribution) (see Figure 4).

Inspiratory efforts during spontaneous breathing, lung volume, owing to different PEEP values, and external airway resistance consistently influenced intraparenchymal gas redistribution. During spontaneous breathing, but not during controlled mechanical ventilation, low lung volume (low PEEP) and high external airway resistances promoted pendelluft, amplifying the initial inspiratory efforts (see Figures 2, 3). Throughout the subsequent flow-dependent inspiratory phase, low PEEP and high external resistances changed the gas-redistribution pattern toward larger areas (gas displacing) of gas redistribution (see Figure 5). Although free from pendelluft, controlled mechanical ventilation was characterized by multifold transients of gas redistribution in the second phase of the inspiration. The loss of diaphragmatic tone can justify the onset of larger areas of intraparenchymal gas redistribution during controlled mechanical ventilation.

### Intraparenchymal Gas Redistribution Before the Onset of Inspiratory Flow (Pendelluft)

Pendelluft was found only during spontaneous breathing (see Figures 2, 3). The inspiratory effort characterizing the early phase of the inspiration was defined by measuring the pressure signal during the time needed to generate a flow at airway opening by overcoming the auto-PEEP (see Table 2). The absence of airway flow at the beginning of the inspiratory efforts was mainly attributable to the auto-PEEP. The negative transpulmonary pressure, generated to counteract the auto-PEEP, induces intrapulmonary gradients of pressure and promotes a redistribution of gas within the lung, independently from the inspiratory flow at the airway opening (Mead et al., 1970).

In our study, the magnitude of pendelluft and its topographical distribution were influenced by PEEP and external airway resistances. Low PEEP (lower than 9 cm H_2_O) as well as high external airway resistances promoted the onset of pendelluft from nondependent (ROI1) to dependent (ROI4) and from cranial (L4) to caudal (L1) regions of the lung (see Figures 2, 3). Although the effects of higher PEEP in reducing pendelluft was already known before (Yoshida et al., 2016b; Morais et al., 2018), we described this event in a three-dimensional manner, showing a cranial-to-caudal gas redistribution and emphasizing the effects of auto-PEEP and inspiratory efforts, as well as of high external resistances on the onset of pendelluft. Promoting auto-PEEP and more intense inspiratory efforts, high external resistances impeded flow at the airway opening. This consequently facilitated gas redistribution within the lung parenchyma. The increase in external resistance is a complication commonly occurring in intubated and mechanically ventilated critically ill patients. High external resistances are indeed easily promoted by small-sized endotracheal tubes and by tracheal secretions (El-Khatib et al., 2008).

During controlled mechanical ventilation, pendelluft did not occur (see Figure 3). This was presumably attributable to the fact that during the initial isovolumetric phase of the inspiration, there was neither active inspiratory efforts nor enough auto-PEEP. Therefore, no forces were acting on heterogeneous portions of the lung to initiate intraparenchymal gas redistribution (pendelluft).

The main advantage of our technique, based on dynamic computed tomography, is the possibility of defining pendelluft by high spatial resolution. Several studies have shown that the pendelluft can be easily diagnosed at bedside by electrical impedance tomography (EIT) (Karagiannidis et al., 2018; Rossi et al., 2019; Santini et al., 2019; Sang et al., 2020). Although presenting a lower spatial resolution than CT, the EIT is a noninvasive, easily repeatable lung-imaging technique preventing exposure to ionizing radiation.

### Intraparenchymal Gas Redistribution During Ongoing Inspiratory Flow

Although the total amount of gas redistribution was comparable between controlled mechanical ventilation and spontaneous breathing (see Figure 4), larger areas of gas redistribution (gas displacing) characterized controlled mechanical ventilation, while smaller and numerous areas (gas scattering) characterized spontaneous breathing. As for the onset of pendelluft, gas redistribution was significantly influenced by the tested ventilatory conditions. PEEP levels higher than 6 cm H_2_O and low external airway resistance facilitated the transition toward gas scattering (see Figure 5). In this late phase of the inspiration, the inspiratory effort was defined by the esophageal pressure and the EAdi peak (see Table 1). High inspiratory effort characterized low PEEP levels. The ratio between the tidal volume and the corresponding EAdi peak during spontaneous breathing, expression of neuroventilatory efficiency, was increased at PEEP levels between 6 and 12 cm H_2_O, owing to an optimal force–length relationship of the diaphragmatic muscular fibers (Evans and Hill, 1914; see Table 1).

Widespread and highly intermingled transients of gas redistribution resulted in a more extended interface and lower gradients of pressure between temporarily inflating and deflating lung regions. This can act as a lung stabilizer and can explain the reduction in atelectasis and the improved oxygenation observed in optimized spontaneous breathing (Putensen et al., 2001; Wrigge et al., 2003; Levine et al., 2008). These findings are well supported by the theory of emergent phenomena (Andersen, 1972; Macklem, 2008) for which continuous and multiple interactions between temporarily inflating and deflating microareas create a macroscopic stability of the entire lung parenchyma, as previously demonstrated in asthma models (Winkler and Suki, 2011; Winkler et al., 2015). Alveolar interdependence and microinstabilities promote macroscopic lung stability (Faffe and Zin, 2009). Alveoli are interconnected in functional structures. Each single unit benefits from mutual support (Mead et al., 1970). These events are even more pronounced in heterogeneously injured lungs in which anisotropic alveolar mechanics is prevalent (Schiller et al., 2001).

One more difference between spontaneous breathing and controlled mechanical ventilation reported in our study was the inversion of the direction of gas redistribution along the cranial–caudal axis (see Figure 5). During spontaneous breathing, paradiaphragmatic regions of the lung (L1) were characterized by smaller areas of gas redistribution (gas scattering) if compared to more cranial regions of the lung (L4). During controlled mechanical ventilation, the lung close to the diaphragm (L1) showed larger areas of gas redistribution (gas displacing) if compared to more cranial regions (L4). During controlled mechanical ventilation, the loss of diaphragmatic tone and its cranial displacement determined paradiaphragmatic atelectasis and loss of alveolar interdependency (Reber et al., 1998), explaining the presence of a larger area of gas redistribution (gas displacing) in the more caudal region.

## Limitations of the Study

The present study has several limitations.

(1) The analysis of gas redistribution reported in the present study is exclusively focused on the inspiratory phase. Further studies are necessary for better characterizing these events over the entire breath.
(2) We studied only continuous positive airway pressure during spontaneous breathing. The choice of continuous positive airway pressure (CPAP) as “ventilatory mode” was due to the will of excluding any interference that a ventilatory support would have had on the spontaneous breathing pattern of the animal. Assisted mechanical ventilation or triggered controlled ventilatory modalities may be characterized by completely different patterns of intraparenchymal gas redistribution.
(3) The current study is based on an animal model of mild ARDS. Swine anatomy substantially differs from human anatomy in terms of diaphragmatic shape, airway displacement, and lung parenchyma. Limitations deriving from anatomical differences should be taken into account when interpreting experimental translational studies. Whether the physiological mechanisms and the pathophysiological implications of these observations could be applied also on patients would require purposely designed clinical studies.
(4) We studied only one specific model of mild ARDS characterized by surfactant depletion. A difference in intraparenchymal gas redistribution among different animal models of lung injury may be hypothesized. Further detailed information about technical aspects of the study has been provided in the Supplementary Material.

## Conclusion

Based on a method characterized by higher spatial and temporal resolution, our study described the complexities of inspiratory gas redistribution events. We showed that pendelluft exclusively characterized spontaneous breathing. Suitable PEEP levels and low external resistances, limiting the inspiration efforts, reduced the onset of pendelluft.

Although “free” from pendelluft, controlled mechanical ventilation promoted large intraparenchymal gas redistribution, affecting interdependency among small functional lung units and threatening lung stability.

## Data Availability Statement

The original contributions presented in the study are included in the article/Supplementary Material, further inquiries can be directed to the corresponding author/s.

## Ethics Statement

The animal study was reviewed and approved by Uppsala regional animal ethics committee (No. C 46_14).

## Author Contributions

MP and GP designed and performed the experiments, measured respiratory mechanics, wrote and applied the programs for data analysis, drafted the manuscript, and revised the manuscript for content. GH and AL designed the experiments, analyzed the data, coordinated the activities, drafted the manuscript, and revised the manuscript for content. All authors contributed to the article and approved the submitted version.

## Conflict of Interest

The authors declare that the research was conducted in the absence of any commercial or financial relationships that could be construed as a potential conflict of interest.
