# Supplementary material for: Inspiratory Efforts, Positive End-Expiratory Pressure, and External Resistances Influence Intraparenchymal Gas Redistribution in Mechanically Ventilated Injured Lungs
Source: Front Physiol. 2021 Feb 9;11:618640. doi: 10.3389/fphys.2020.618640 (PMC7900494; doi:10.3389/fphys.2020.618640)
Supplement: Supplementary file 1 [file Image_1.PDF]

Supplementary material

Inspiratory efforts, positive end-expiratory pressure and external resistances influence intra-parenchymal gas redistribution in mechanically ventilated injured lungs.

Mariangela Pellegrini, M.D., Ph.D. [a,b]; Göran Hedenstierna, M.D., Ph.D [c]; Anders Larsson, M.D., Ph.D [a]; Gaetano Perchiazzi, M.D., Ph.D [a,b]

a. *Hedenstierna Laboratory, Department of Surgical Sciences, Uppsala University, Uppsala, Sweden*
b. *Intensive Care Unit, Department of Anesthesia, Operation and Intensive Care, Uppsala University Hospital, Uppsala, Sweden*
c. *Hedenstierna Laboratory, Department of Medical Sciences, Uppsala University, Uppsala, Sweden*

Methods

The study was approved by the Regional Animal Ethics Committee (No. C 46_14) and was performed at the Hedenstierna Laboratory (Uppsala University, Sweden). The animals were treated in adherence with the European Union Directive 2010/63/EU for animal experiments and according to the National Institutes of Health Guidelines for the use and care of animals. The experimental protocol was developed according to the principles expressed in the PREPARE guidelines (Smith et al., 2018) and reported according to the ARRIVE guidelines (Kilkenny et al., 2010). Six anesthetized animals (Yorkshire breed pigs, mean weight ± SEM of 29.9 ± 2.6 kg) underwent repeated lung lavages to create a mild acute respiratory distress syndrome (ARDS) model.

Animal anesthesia and instrumentation

The animals were premedicated with intramuscular tiletamine–zolazepam (6 mg/kg, Boeringer Ingelheim, Copenhagen, Denmark) and xylazine (2.2 mg/kg, Rompun Vet., Bayer Animal Health GmbH, Leverkusen, Germany). They were positioned supine and anesthetized, thus ensuring continuous and spontaneous respiratory activity. To preserve the respiratory drive activity, anesthesia was induced by a continuous infusion of ketamine (20 mg/kg/h, ketaminol; Vetpharma, Zurich, Switzerland) via an 18 G catheter placed in an ear vein. Low doses (0.6–1.2 mg/kg/h) propofol (B. Braun Melsungen AG, Melsungen, Germany) were infused in the case of animal–ventilator asynchronies. Remifentanil, 0.25–0.5 mcg/kg/min (Remifentanil Orion, Orion Pharma, Orionintie 1, Espoo, Finland), was continuously infused during periods of the protocol when spontaneous breathing suppression was needed. Both during lung lavage procedures and the phase of controlled mechanical ventilations, complete muscle relaxation was induced by intravenous bolus injections of 20-mg rocuronium (Rocuronium Fresenius Kabi 10 mg/mL, Fresenius Kabi AB, Uppsala, Sweden). After supplemental local anesthesia, an endotracheal tube (n. 9, Mallinckrodt Pharmaceuticals, Athlone, Ireland) was positioned via surgical tracheotomy and connected

to the ventilator (Servo-I ventilator, Maquet-Getinge Critical Care, Solna, Sweden). The starting ventilatory mode was pressure support ventilation with a positive end-expiratory pressure (PEEP) of 5 cmH_2O, an inspiratory pressure (Pinsp) of 10 cmH_2O, and an inspiratory oxygen fraction (FIO_2) of 0.5. Using an ultrasound-guided procedure, a double-lumen central venous catheter (percutaneous sheath introducer kit, Exacta, Argon Medical Devices, Singapore) was inserted into the femoral vein. A flow-directed Swan-Ganz pulmonary artery catheter (PAC; 7.0 French, Swan-Ganz thermodilution catheter, Baxter, Irvine, CA, USA) was introduced through the femoral central venous catheter in the main venous systemic system throughout the vena cava inferior to the right heart. By pressure monitoring, the catheter tip was positioned in the pulmonary artery. The PAC and a peripheral artery catheter placed in the femoral artery were used for hemodynamic monitoring. Arterial blood was collected for each studied condition and analyzed by an ABL 800 flex (Radiometer, Copenhagen, Denmark). The heart rate (HR) and transcutaneous oxygen saturation by pulse oximetry (SpO_2) were continuously monitored (SC 9000 XL, Siemens Medical Systems Inc., Danvers, MA, USA). The urinary output was measured via a surgically inserted suprapubic urinary catheter. The maintenance fluid therapy comprised 0.9% sodium chloride (infusion rate of 10 mL/kg/h) IV. Neither inotropic support nor extra fluid boluses were needed.

Respiratory mechanics

Gastric and esophageal balloon catheters (Esophageal Catheter, Erich Jaeger GmbH, Höchberg, Germany) were placed, and their position was optimized by the occlusion test method described by Baydur and coworkers (Baydur et al., 1982). In this way, both esophageal (Pes) and gastric pressures (Pga) were continuously recorded. At the same time, airway pressure (Paw) and flow (V'aw) were continuously measured at the airway opening. V'aw was detected by a Fleisch pneumotachograph (Laminar Flow Element type PT, Special Instruments GmbH, Nördlingen, Germany) positioned between the endotracheal tube and the Y-piece and connected to a differential pressure transducer (Diff-Cap Pressure Transducer, Special Instruments GmbH, Nördlingen, Germany). Pes, Pga, and Paw were directly detected by pressure transducers (DigimaClic Pressure Transducers, Special Instruments GmbH, Nördlingen, Germany). An

analog-to-digital converter card (DAQ-card AI-16XE50, National Instruments Corp., Austin, TX, USA) received all the aforementioned signals, which were then stored at a sampling frequency of 200 Hz on a personal computer (Intel Centrino, Intel Corp., Santa Clara, CA, USA) using the BioBench Software (ver. 1.0, National Instruments Corp., Austin, TX, USA). In addition, the electrical diaphragmatic (EAdi) signal was continuously recorded at the level of the diaphragmatic dome using a 16 F nasogastric feeding tube equipped with multiple array electrodes (Maquet-Getinge Critical Care, Solna, Sweden) at the gastro-esophageal junction. The EAdi catheter was connected to the neutrally adjusted ventilatory assistance (NAVA) module on the Servo-I ventilator and, through the latter by means of a serial cable, to a personal computer (Intel Centrino, Intel Corp., Santa Clara, CA, USA) equipped with the Servo-tracker V 4.0 software (Maquet-Getinge Critical Care, Solna, Sweden). The EAdi signal was sampled at 100 Hz. After the experimental part and before EAdi signal analysis, the effects of the low-pass digital filter (applied by NAVA software for noise reduction) were removed by a purposely written MATLAB script. Further detailed information about the optimization of the EAdi catheter positioning and the process of removing the effects of the EAdi filters characterizing the NAVA software can be found in the main text and supplementary material of our previously published article (Pellegrini et al., 2017).

Lung injury

A model of surfactant-deficient mild ARDS and lung collapse was obtained. To create lung injury, lung lavages (30 ml/kg of warmed isotonic saline at 37 °C) were repeated until a stable ratio between the partial pressure of arterial oxygen and inspired oxygen fraction (PaO_2/FIO_2) of 250 was established at a PEEP of 5 cmH_2O.

Computed tomography (CT) protocol

After lung injury, under stabilized conditions, the animals were transferred to the CT facility and positioned supine on the CT table (64-slices CT Somatom Definition; Siemens AG, Erlangen, Germany). Two lung scanograms were acquired during anesthesia and muscle relaxation at PEEP levels of 15 and 0

cmH_2O. These scanograms allowed for the estimation of the maximal displacement of both the diaphragm and EAdi catheter between the two tested extreme PEEP levels. Then, the infusion of the muscle relaxant was discontinued, and the animals regained a full spontaneous respiratory drive. The animals started breathing under continuous positive airway pressure (CPAP) at a PEEP of 0 cmH_2O. The PEEP was then gradually increased up to 15 cmH_2O. Two different external airway resistances were tested during spontaneous breathing: a low external resistance (SB-LowR) achieved by an endotracheal tube with an internal diameter of 9 mm and a high external resistance (SB-HighR) achieved by an endotracheal tube with an internal diameter of 6 mm.

CT images were acquired during ongoing spontaneous breathing in dynamic conditions at six PEEP levels (15, 12, 9, 6, 3, and 0 cmH_2O). At each PEEP level, stable ventilation for at least 5 min was assured before image acquisition. CT scans were acquired for 10 s, with an acquisition rate of 20 Hz (0.05-s time interval between consecutive images). Two separate 5-mm-thick slices were simultaneously acquired at 1 (L1) and 4 cm (L4), respectively, from the diaphragmatic dome. CT voxel dimensions were 5 mm × 0.5 mm × 0.5 mm. After CT acquisition, during spontaneous breathing, the animals were muscle relaxed and underwent a recruitment maneuver. The same CT protocol described above was repeated during controlled mechanical ventilation, starting from a PEEP of 15 cmH_2O. Pressure-controlled ventilation was applied by selecting appropriate inspiratory pressure (Pinsp) and respiratory rate values to achieve ventilation comparable to that obtained during the preceding period of spontaneous breathing. Complete suppression of the EAdi signal was ensured during controlled mechanical ventilation by muscle paralysis.

<u>CT image analysis</u>

The collected CT images were processed as two-dimensional square matrices (512 × 512 voxels). The CT voxel dimensions were 5 mm × 0.5 mm × 0.5 mm. The train of tidal volumes derived from the subsequent CT images allowed for the synchronization between CT images and spirometric data. The images acquired during the inspiratory phases were selected for further analysis. The inspiratory phase was defined as the portion of the breath between the early deflection of Pes (during spontaneous breathing) or Paw (during

controlled mechanical ventilation) and the inspiratory flow peak at the airway opening. The selected images were processed using semiautomatic lung parenchyma border delineation. Big vessels, the heart, and mediastinal structures were all excluded from the analysis. Each CT image provided information about the volume of gas inside the lung parenchyma. Delta-volume images were obtained to allow for the study of the regional flow distribution. Although two consecutively acquired CT images were only 0.05 s apart and the structural differences between them were minimal, a direct superimposition of the two original consecutive images could have caused subtraction errors between mismatched areas. Thus, an image registration (IR) procedure, normally used for the image processing of medical images (Pennec et al., 1999; Hill et al., 2001; Guerrero et al., 2006; Gu et al., 2010; Kaczka et al., 2011; Perchiazzi et al., 2014) was applied.

Pendelluft analysis

Pendelluft can characterize the early isovolumetric phase of inspiration, when the inspiratory effort is not yet coupled with an inspiratory gas flow from outside the lungs. During this phase, the internal redistribution of gas is possible. The time related to auto-PEEP was calculated to estimate the time of isovolumetric inspiration (see *Figure 1*). During spontaneous breathing, dynamic auto-PEEP was calculated as the difference in Pes between the point of zero flow at end-expiration and the onset of the subsequent inspiratory flow. During controlled mechanical ventilation, auto-PEEP was estimated by calculating the delta in airway pressure between end-expiration and the onset of inspiratory flow (Rossi et al., 1995). Based on the longest recorded time related to auto-PEEP, the number of CT images at the beginning of the inspiration to be included in the pendelluft analysis was determined. Each delta-volume image was then divided into four equally sized regions of interest (ROIs; ROI1, ROI2, ROI3, and ROI4), with ROI1 being the most non-dependent and ROI4 the most dependent one. Gas flow [mL / 0.05 seconds] normalized to its extension [mL / 0.05 seconds / mm^3] were computed for each ROI.

Local gas redistribution during ongoing inspiration

Temporal sequences of gas redistribution throughout the inspiratory phase have been inspected by creating trains of delta-volume images (see *supplementary video 1–6*).

The quadtree decomposition (QtD) algorithm was applied to the delta-volume images and was used for analyzing the gas-redistribution pattern during ongoing inspiratory flow. The QtD is a standard algorithm generally used in image processing (Rhee et al., 2000; Thompson and Shure, 2007). It iteratively divides square images into four equal squares until a predetermined criterion of homogeneity is satisfied. If the homogeneity criterion is not satisfied, the analyzed image portion is further divided into four squares. Consequently, the mean area of the square (AreaSq) expressed in cm^2 reveals the degree of heterogeneity: the smaller the AreaSq, the higher the heterogeneity (see *Figure 2*). In the current study, the criterion of homogeneity is defined as a flow variation among the contiguous areas of the same selected square equal to or lower than 10%. Considering that the regional delta volume per voxel was within the range of $[-1 * 10^{-4}, +1 * 10^{-4}]$ mL, a difference in volume equal to or lower than $2 * 10^{-5}$ mL was considered as homogeneous. Moreover, AreaSq equal to 2 cm^2 was arbitrarily chosen as a limit to distinguish between two patterns. For AreaSq equal to or smaller than 2 cm^2, the pattern was defined as gas scattering; for AreaSq greater than 2 cm^2, the pattern was defined as gas displacing.

Statistics

Data analysis and statistical tests were performed using dedicated MATLAB scripts (MATLAB and Statistics Toolbox Release 2019b). All the variables were tested for normal distribution, confirmed by a one-sample Kolmogorov–Smirnov test ($\alpha = 0.05$). The analysis of variance method was used to test statistical differences. A two-sample Student t-test was used for paired comparisons. Bonferroni’s correction for multiple comparisons was applied when needed. Descriptive statistics were reported using mean (± SEM).

Technical aspects

Image registration (IR) was used to obtain delta volume from the subtraction of two consecutively acquired CT images. The IR procedure aims to overlay different images of the same object that have been obtained at different times, from different viewpoints, or by different sensors. It is commonly used for image processing in several medical fields because it allows for comparisons among images (Pennec et al., 1999; Hill et al., 2001). In the present study, IR has been applied to pairs of consecutive CT images acquired at intervals of 0.05 s. This extremely brief duration between two acquisitions implied small volume increments and minimal displacements of lung structures. The non-rigid registration approach used in this study has been already applied on lung images by Gu and coworkers (Gu et al., 2010).

Pendelluft analysis assumes a complete absence of inspiratory flow in the airway opening during the isovolumetric phase. Thus, it is important to consider that during the expiratory phase, the Servo-I ventilator in the CPAP mode has both inspiratory and expiratory valves open and maintains a constant flow of 2 L/m between them. When an inspiratory phase is triggered, the ventilator generates additional flow to increase airway pressure and ventilate the patient. The onset of this “ventilatory” flow has a delay of about 30 ms from the triggering (personal communication, Magnus Hallbäck, Maquet-Getinge, Solna, Sweden). In the present study, we observed a maximal isovolumetric time (defined as the delay time between the onset of inspiratory effort and the onset of inspiratory flow) of 150 ms. Although the presence of auto-PEEP can justify this delay, we cannot ignore that the first 30-ms delay can be attributable to the abovementioned delay by ventilator.

The dynamic auto-PEEP estimated during spontaneous breathing assumes the complete relaxation of the abdominal expiratory muscles. In the case of active contraction of the expiratory muscles during expiration, the initial inspiratory efforts will combine with the relaxation of the expiratory muscles. In this case, the calculated dynamic auto-PEEP could overestimate the real auto-PEEP (Zakynthinos et al., 2000; Akoumianaki et al., 2014). In the present study, the calculated dynamic auto-PEEP values were low (the highest value recorded at a PEEP of 0 cmH_2O and high external resistance was 2.02 cmH_2O); thus, the risk of overestimation was minimal. During controlled mechanical ventilation, the auto-PEEP estimated using dynamic techniques (Rossi et al., 1995) reflects the lowest value of regional dynamic auto-PEEP. In

this case, there is a risk of underestimation of the real static auto-PEEP. The rationale behind this is that in dynamic conditions, the heterogeneous multiple compartments characterizing injured lungs have probably not reached equilibrium (Maltais et al., 1994; Brochard, 2002). However, in the present study, the auto-PEEP calculation was finalized to the estimation of the no-flow time at the beginning of the inspiratory phase to determine the number of CT images (and corresponding delta volumes) needed for the pendelluft analysis.

References

Akoumianaki, E., Maggiore, S. M., Valenza, F., Bellani, G., Jubran, A., Loring, S. H., et al. (2014). The application of esophageal pressure measurement in patients with respiratory failure. *Am. J. Respir. Crit. Care Med.* 189, 520–31. doi:10.1164/rccm.201312-2193CI.

Baydur, A., Behrakis, P., Zin, W., Jaeger, M., and Milic-Emili, J. (1982). A simple method for assessing the validity of the esophageal balloon technique. *Am Rev Respir Dis* 126, 788–791.

Brochard, L. (2002). Intrinsic (or auto-) PEEP during controlled mechanical ventilation. *Intensive Care Med.* 28, 1376–1378. doi:10.1007/s00134-002-1438-8.

Gu, X., Pan, H., Liang, Y., Castillo, R., Yang, D., Choi, D., et al. (2010). Implementation and evaluation of various demons deformable image registration algorithms on a GPU. *Phys. Med. Biol.* 55, 207–19. doi:10.1088/0031-9155/55/1/012.

Guerrero, T., Castillo, R., Sanders, K., Price, R., Komaki, R., and Cody, D. (2006). Novel method to calculate pulmonary compliance images in rodents from computed tomography acquired at constant pressures. *Phys. Med. Biol.* 51, 1101–12. doi:10.1088/0031-9155/51/5/003.

Hill, D. L. G., Batchelor, P. G., Holden, M., and Hawkes, D. J. (2001). Medical image registration. *Phys. Med. Biol.* 46, R1–R45. doi:10.1088/0031-9155/46/3/201.

Kaczka, D. W., Cao, K., Christensen, G. E., Bates, J. H. T., and Simon, B. a (2011). Analysis of regional mechanics in canine lung injury using forced oscillations and 3D image registration. *Ann. Biomed. Eng.* 39, 1112–24. doi:10.1007/s10439-010-0214-0.

Kilkenny, C., Browne, W. J., Cuthill, I. C., Emerson, M., and Altman, D. G. (2010). Improving bioscience research reporting: the ARRIVE guidelines for reporting animal research. *PLoS Biol.* 8, e1000412. doi:10.1371/journal.pbio.1000412.

Maltais, F., Reissmann, H., Navalesi, P., Hernandez, P., Gursahaney, A., Ranieri, V., et al. (1994). Comparison of Static and Dynamic Measurements of Intrinsic PEEP in Mechanically Ventilated

Patients. *Am. J. Respir. Crit. Care Med.* 150, 1318–1324.

Pellegrini, M., Hedenstierna, G., Roneus, A., Segelsjö, M., Larsson, A., and Perchiazzi, G. (2017). The diaphragm acts as a brake during expiration to prevent lung collapse. *Am. J. Respir. Crit. Care Med.* 195. doi:10.1164/rccm.201605-0992OC.

Pennec, X., Cachier, P., and Ayache, N. (1999). Understanding the "Demon's algorithm": 3D non-rigid registration by gradient descent. in *2nd Int. Conf. on Medical Image Computing and Computer-Assisted Intervention (MICCAI '99)* (Cambridge), 597–605.

Perchiazzi, G., Rylander, C., Derosa, S., Pellegrini, M., Pitagora, L., Polieri, D., et al. (2014). Regional distribution of lung compliance by image analysis of computed tomograms. *Respir. Physiol. Neurobiol.* 201, 60–70. doi:10.1016/j.resp.2014.07.001.

Rhee, I., Martin, G. R., Muthukrishnan, S., and Packwood, R. a. (2000). Quadtree-structured variable-size block-matching motion estimation with minimal error. *IEEE Trans. Circuits Syst. Video Technol.* 10, 42–50. doi:10.1109/76.825857.

Rossi, A., Polese, G., Brandi, G., and Conti, G. (1995). Intrinsic positive end-expiratory pressure (PEEPi). *Intensive Care Med.* 21, 522–536. doi:10.1007/BF01706208.

Smith, A. J., Clutton, R. E., Lilley, E., Hansen, K. E. A., and Brattelid, T. (2018). PREPARE: guidelines for planning animal research and testing. *Lab. Anim.* 52, 135–141. doi:10.1177/0023677217724823.

Thompson, C., and Shure, L. (2007). "Analyzing an image: using quadtree decomposition," in *Image Processing Toolbox, for version 5.4. The MathWorks, Natick*, 11.21-11.23.

Zakynthinos, S. G., Vassilakopoulos, T., Zakynthinos, E., Mavrommatis, a, and Roussos, C. (2000). Contribution of expiratory muscle pressure to dynamic intrinsic positive end-expiratory pressure: validation using the Campbell diagram. *Am. J. Respir. Crit. Care Med.* 162, 1633–40. doi:10.1164/ajrccm.162.5.9903084.

Inspiratory efforts, positive end-expiratory pressure and external resistances influence intra-parenchymal gas redistribution in mechanically ventilated injured lungs.

Mariangela Pellegrini, M.D., Ph.D; Göran Hedenstierna, M.D., Ph.D; Anders Larsson, M.D., Ph.D; Gaetano Perchiazzi, M.D., Ph.D

Supplementary Figures and Tables

Figure E1

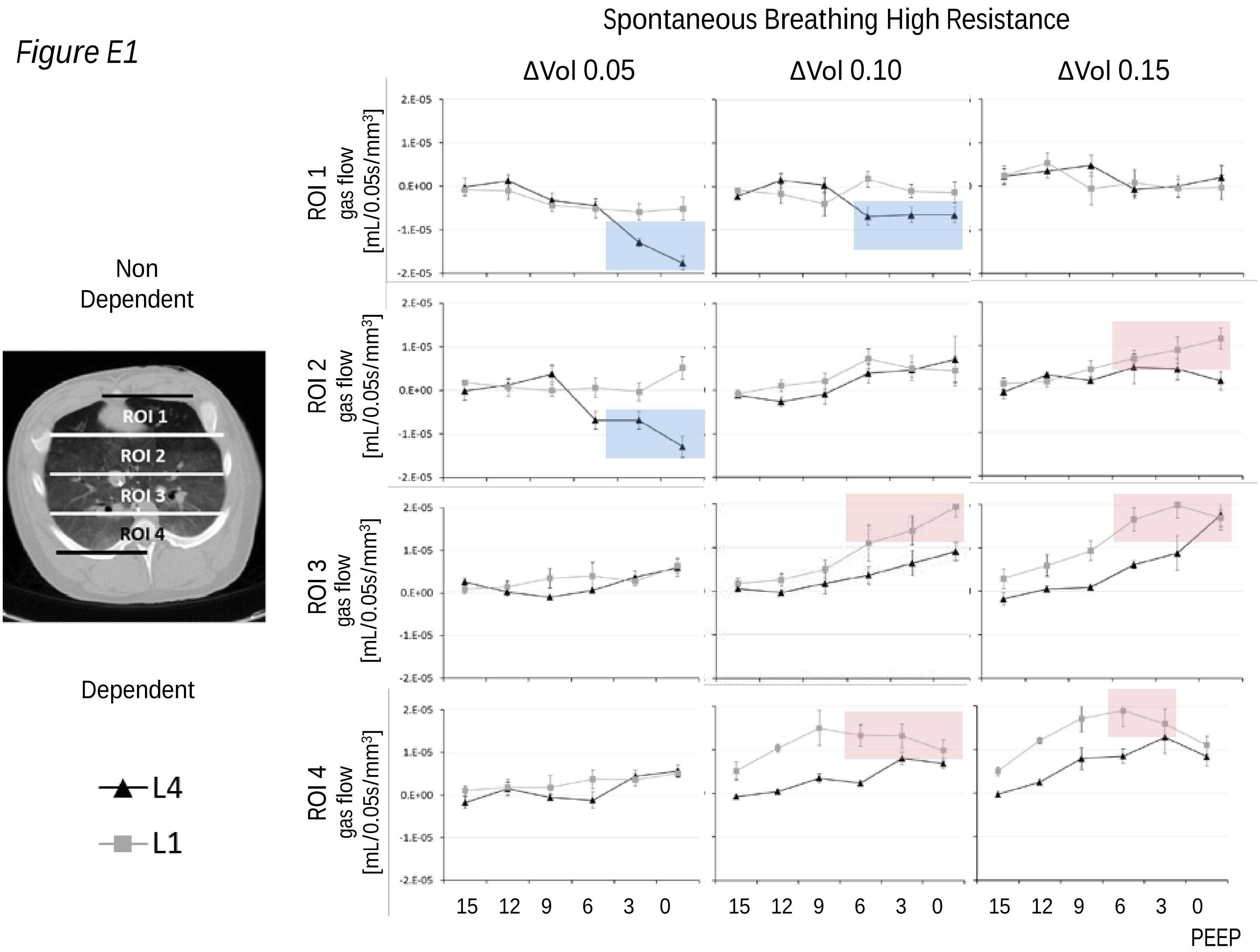

Spontaneous Breathing High Resistance
ΔVol 0.05
ΔVol 0.10
ΔVol 0.15
Non
Dependent
Dependent
ROI 1
ROI 2
ROI 3
ROI 4
gas flow
[mL/0.05s/mm³]
2.E-05
1.E-05
0.E+00
-1.E-05
-2.E-05
15
12
9
6
3
0
PEEP
L4
L1

***Figure E1*. Gas redistribution during the isovolumetric phase of inspiration: the pendelluft analysis. Pendelluft during spontaneous breathing and high external airway resistance.**
Complementary information in Figure 2 and Figure 3.

Based on the estimated time to the onset of the inspiratory flow at the beginning of the inspiration, three delta volumes at respectively 0.05, 0.10, and 0.15 s (ΔVol0.05, ΔVol0.10, and ΔVol0.15) were analyzed. Each delta-volume image was divided into four regions of interest (ROIs) along the gravitational axis (ROI1, ROI2, ROI3, and ROI4).
The total amount of gas redistribution normalized for the corresponding ROI volume [mL/mm^3; mean ± SE] (y-axis) was calculated at six different positive end-expiratory pressure (PEEP) levels (x-axis). The gray line indicates the distance of 1 cm from the diaphragmatic dome (L1), and the black line indicates the distance of 4 cm from the diaphragmatic dome (L4).

SB-LowR L1

PEEP levels		ΔVol 0.05				ΔVol 0.10				ΔVol 0.15			
		ROI1	ROI2	ROI3	ROI4	ROI1	ROI2	ROI3	ROI4	ROI1	ROI2	ROI3	ROI4
15	12	0.61	0.90	0.88	0.69	0.52	0.76	0.15	0.31	0.68	0.26	0.12	0.23
15	9	0.99	0.70	0.51	0.02 *	0.17	0.16	0.02 *	0.18	0.73	0.10	0.01 *	0.06
15	6	0.24	0.17	0.50	0.01 *	0.80	0.06	0.01 *	0.10	0.29	0.01 *	0.01 *	0.01 *
15	3	0.08	0.01	0.55	0.01 *	0.76	0.01 *	0.01 *	0.04 *	0.68	<0.01 *	<0.01 *	0.03 *
15	0	0.81	0.03 *	0.37	0.01 *	0.77	0.01 *	<0.01 *	0.01 *	0.33	<0.01 *	<0.01 *	0.03 *
12	9	0.61	0.69	0.55	0.03 *	0.27	0.27	0.4	0.06	0.95	0.32	0.31	0.39
12	6	0.26	0.03 *	0.55	0.01 *	0.53	0.13	0.02 *	0.52	0.63	0.02 *	0.07	0.15
12	3	0.01 *	0.00 *	0.61	0.01 *	0.15	0.01 *	0.02 *	0.33	0.97	<0.01 *	0.01 *	0.25
12	0	<0.01 *	0.01 *	0.39	0.01 *	0.15	0.02 *	0.01 *	0.69	0.65	<0.01 *	0.01 *	0.49
9	6	0.24	0.28	0.82	0.83	0.18	0.72	0.73	0.93	0.59	0.41	0.71	0.60
9	3	0.08	0.02 *	0.84	0.60	0.51	0.08	0.27	0.85	0.98	0.02 *	0.33	0.81
9	0	0.80	0.04 *	0.99	0.65	0.5	0.07	0.17	0.76	0.61	0.02 *	0.16	0.74
6	3	0.65	0.02 *	0.99	0.68	0.65	0.12	0.44	0.71	0.54	0.03 *	0.44	0.77
6	0	0.48	0.08	0.74	0.76	0.67	0.10	0.29	0.77	1.00	0.03 *	0.19	0.33
3	0	0.09	0.56	0.79	0.85	0.99	0.65	0.79	0.50	0.57	0.51	0.41	0.52

SB-HighR L1

PEEP levels		ΔVol 0.05				ΔVol 0.10				ΔVol 0.15			
		ROI1	ROI2	ROI3	ROI4	ROI1	ROI2	ROI3	ROI4	ROI1	ROI2	ROI3	ROI4
15	12	0.88	0.24	0.48	0.58	0.96	0.32	0.87	0.04 *	0.75	0.77	0.64	0.04 *
15	9	0.04 *	0.22	0.31	0.55	0.36	0.15	0.03 *	0.04 *	0.96	0.28	0.05	0.04 *
15	6	0.02 *	0.27	0.34	0.27	0.90	0.03 *	0.04 *	0.04 *	0.50	0.04 *	0.04 *	0.01 *
15	3	0.04 *	0.66	0.31	0.10	0.86	0.04 *	0.05 *	0.04 *	0.56	0.02 *	0.05 *	0.08
15	0	0.05 *	0.05 *	0.04 *	0.05 *	0.9	0.16	0.01 *	0.05 *	0.56	0.01 *	0.02 *	0.05
12	9	0.71	0.86	0.62	0.89	0.43	0.49	0.42	0.46	0.92	0.37	0.56	0.90
12	6	0.01 *	0.94	0.59	0.54	0.90	0.14	0.18	0.34	0.74	0.04 *	0.20	0.31
12	3	0.04 *	0.56	0.78	0.30	0.89	0.12	0.04 *	0.12	0.49	0.02 *	0.07	0.34
12	0	0.04 *	0.02 *	0.15	0.18	0.90	0.30	0.04 *	0.12	0.49	0.01 *	0.04 *	0.26
9	6	0.22	0.93	0.88	0.68	0.58	0.31	0.37	0.78	0.75	0.36	0.33	0.22
9	3	0.51	0.61	0.77	0.46	0.44	0.30	0.15	0.33	0.79	0.38	0.13	0.27
9	0	0.84	0.02 *	0.39	0.23	0.65	0.53	0.02 *	0.30	0.78	0.33	0.08	0.19
6	3	0.83	0.61	0.69	0.77	0.97	0.94	0.63	0.49	0.32	0.59	0.57	0.83
6	0	0.44	0.02 *	0.58	0.62	0.98	0.79	0.02 *	0.43	0.32	0.63	0.71	0.72
3	0	0.67	0.01 *	0.19	0.81	0.96	0.82	0.48	0.86	0.96	0.84	0.76	0.95

MV L1

PEEP levels		ΔVol 0.05				ΔVol 0.10				ΔVol 0.15			
		ROI1	ROI2	ROI3	ROI4	ROI1	ROI2	ROI3	ROI4	ROI1	ROI2	ROI3	ROI4
15	12	0.37	0.46	0.94	0.62	0.73	0.78	0.25	0.28	0.46	0.42	0.20	0.35
15	9	0.89	0.72	0.91	0.49	0.05 *	0.89	0.66	0.85	0.83	0.22	0.27	0.99
15	6	0.83	0.97	0.46	0.94	0.02 *	0.29	0.28	0.66	0.87	0.01	0.11	0.94
15	3	0.12	0.43	0.88	0.89	0.07	0.23	0.09	0.61	0.04 *	0.04 *	0.14	0.62
15	0	0.56	0.07	0.27	0.93	0.04 *	0.11	0.06	0.63	0.85	0.01 *	0.01	0.31
12	9	0.41	0.57	0.88	0.89	0.15	0.85	0.51	0.11	0.61	0.81	0.67	0.28
12	6	0.43	0.36	0.56	0.61	0.12	0.70	0.59	0.05	0.59	0.30	0.73	0.20
12	3	0.46	0.89	0.86	0.57	0.24	0.57	0.38	0.05	0.07	0.11	0.83	0.09
12	0	0.57	0.37	0.54	0.53	0.19	0.46	0.61	0.05	0.45	0.11	0.24	0.03 *
9	6	0.93	0.56	0.55	0.44	0.81	0.37	0.77	0.60	0.96	0.34	0.87	0.94
9	3	0.12	0.52	0.93	0.31	0.61	0.29	0.09	0.50	0.06	0.12	0.51	0.52
9	0	0.63	0.02	0.26	0.28	0.59	0.15	0.18	0.54	0.73	0.12	0.07	0.19
6	3	0.13	0.35	0.75	0.94	0.68	0.77	0.06	0.69	0.06	0.19	0.54	0.41
6	0	0.69	0.06	0.09	0.99	0.65	0.56	0.11	0.80	0.76	0.16	0.05	0.07
3	0	0.16	0.57	0.43	0.74	0.98	0.84	0.55	0.95	0.08	0.46	0.36	0.22

***Table E1*. Pendelluft analysis.**
Statistical differences among the six studied PEEP levels, at L1.
ANOVA (α=0.05) with Bonferroni's correction for multiple comparisons.
***** to mark statistical differences.
L1 is the layer at 1 cm from the diaphragmatic dome.

ΔVol 0.05 ΔVol 0.10 and ΔVol 0.15 are respectively the delta volumes considered at 0.05, 0.10 and 0.15 seconds.
SB-LowR: spontaneous breathing at low airway resistances;
SB-HIghR: spontaneous breathing at high airway resistances;
MV: mechanical ventilation.

SB-LowR L4

PEEP levels		ΔVol 0.05				ΔVol 0.10				ΔVol 0.15			
		ROI1	ROI2	ROI3	ROI4	ROI1	ROI2	ROI3	ROI4	ROI1	ROI2	ROI3	ROI4
15	12	0.93	0.90	0.67	0.47	0.77	0.25	0.37	0.57	0.19	0.11	0.14	0.06
15	9	0.76	0.18	0.54	0.02 *	0.00 *	0.06	0.01 *	0.53	0.53	0.01 *	0.07	0.05
15	6	0.50	0.81	0.63	0.47	0.04 *	0.31	0.01 *	0.06	0.51	0.01 *	0.20	0.05
15	3	0.02 *	0.04 *	0.51	0.05	0.02 *	0.00 *	0.01 *	<0.01 *	<0.01 *	0.01 *	<0.01 *	0.01 *
15	0	0.02 *	0.04 *	0.01 *	0.01 *	<0.01 *	<0.01 *	0.01 *	<0.01 *	<0.01 *	<0.01 *	<0.01 *	0.01 *
12	9	0.83	0.19	0.77	0.20	0.03 *	0.30	<0.01 *	0.15	0.73	0.22	0.77	0.12
12	6	0.76	0.69	0.90	0.83	0.04 *	0.88	0.01 *	0.12	0.55	0.98	0.51	0.39
12	3	0.02 *	0.02 *	0.01 *	0.01 *	0.04 *	0.01 *	0.01 *	0.01 *	0.01 *	0.86	<0.01 *	0.03 *
12	0	0.01 *	0.46	0.01 *	0.02 *	0.02 *	0.01 *	<0.01 *	0.01 *	0.01 *	0.06	<0.01 *	0.06
9	6	0.86	0.13	0.88	0.52	0.01 *	0.55	0.29	0.91	0.91	0.22	0.61	0.48
9	3	0.02 *	0.01 *	0.03 *	0.02 *	0.01 *	0.03 *	0.12	0.03 *	0.01 *	0.34	<0.01 *	0.31
9	0	0.01 *	0.11	0.01 *	0.04 *	<0.01 *	0.01 *	0.09 *	0.04 *	0.01 *	0.26	<0.01 *	0.71
6	3	0.01 *	0.03 *	0.02 *	0.05	0.86	0.03 *	0.43	0.02 *	0.01 *	0.85	0.16	0.13
6	0	0.65	0.66	0.01 *	0.04 *	0.65	0.03 *	0.02 *	0.02 *	0.33	0.06	0.07	0.29
3	0	0.42	0.19	0.25	0.52	0.48	0.28	0.95	0.41	0.78	0.10	0.23	0.47

SB-HighR L4

PEEP levels		ΔVol 0.05				ΔVol 0.10				ΔVol 0.15			
		ROI1	ROI2	ROI3	ROI4	ROI1	ROI2	ROI3	ROI4	ROI1	ROI2	ROI3	ROI4
15	12	0.67	0.44	0.92	0.47	0.15	0.17	0.62	0.70	0.32	0.33	0.71	0.29
15	9	0.06	0.28	0.34	0.59	0.36	0.96	0.87	0.66	0.67	0.50	0.44	0.04 *
15	6	0.59	0.20	0.10	0.52	0.01 *	0.01 *	0.01 *	0.20	0.85	0.59	<0.01 *	0.04 *
15	3	0.04 *	0.12	0.06	0.11	0.01 *	0.03 *	0.04 *	0.02 *	0.94	0.49	0.03 *	0.01 *
15	0	0.04 *	0.01 *	0.07	0.05 *	0 *	0.03 *	0.04 *	<0.01 *	0.91	0.31	0.02 *	0.03 *
12	9	0.28	0.70	0.42	0.24	0.82	0.54	0.94	0.90	0.55	0.70	0.60	0.25
12	6	0.37	0.73	0.14	0.24	0.01 *	0.04 *	0.05 *	0.40	0.49	0.99	<0.01 *	0.01 *
12	3	0.04 *	0.29	0.06	0.06	0.01 *	0.01 *	0.05 *	0.04 *	0.34	0.94	0.04 *	<0.01 *
12	0	0.04 *	0.03 *	0.01 *	0.03 *	<0.01 *	0.01 *	0.02 *	0.00 *	0.45	0.87	0.02 *	0.01 *
9	6	0.11	0.86	0.52	0.78	0.60	0.27	0.23	0.60	0.86	0.85	<0.01 *	0.48
9	3	0.01 *	0.45	0.11	0.15	0.42	0.01 *	0.11	0.09	0.65	0.84	0.04 *	0.14
9	0	0.02 *	0.04 *	0.03 *	0.04 *	0.43	0.03 *	0.03 *	0.01 *	0.80	0.62	0.02 *	0.18
6	3	0.02 *	0.32	0.20	0.31	0.83	0.49	0.46	0.07	0.81	0.97	0.37	0.17
6	0	0.31	0.28	0.05 *	0.02 *	0.22	0.90	0.35	0.01 *	0.95	0.92	0.10	0.27
3	0	0.60	0.89	0.57	0.69	0.12	0.69	0.91	0.13	0.87	0.85	0.33	0.80

MV L4

PEEP levels		ΔVol 0.05				ΔVol 0.10				ΔVol 0.15			
		ROI1	ROI2	ROI3	ROI4	ROI1	ROI2	ROI3	ROI4	ROI1	ROI2	ROI3	ROI4
15	12	0.45	0.37	0.62	0.90	0.99	0.55	0.78	0.79	0.59	0.39	0.03	0.79
15	9	0.36	0.33	0.74	0.51	0.53	1.00	0.38	0.45	0.47	0.68	0.05	0.78
15	6	0.56	0.80	0.96	0.70	0.28	0.79	0.61	0.53	0.93	0.90	0.02 *	0.87
15	3	0.03 *	1.00	0.45	0.36	0.10	0.63	0.05	0.37	0.02 *	0.01 *	<0.01 *	0.44
15	0	0.03 *	0.20	0.53	0.48	0.87	0.86	0.20	0.17	0.02 *	0.04 *	<0.01 *	0.78
12	9	0.11	0.12	0.47	0.74	0.48	0.10	0.67	0.66	0.95	0.53	0.56	0.62
12	6	0.12	0.29	0.52	0.87	0.23	0.35	0.93	0.83	0.73	0.50	0.35	0.91
12	3	0.04 *	0.45	0.18	0.70	0.09	0.06	0.10	0.68	0.43	0.03 *	0.01 *	0.72
12	0	0.04 *	0.59	0.22	0.80	0.86	0.38	0.3	0.40	0.33	0.07	0.05 *	0.61
9	6	0.60	0.47	0.74	0.83	0.66	0.71	0.62	0.71	0.67	0.89	0.18	0.66
9	3	0.83	0.40	0.87	0.99	0.20	0.18	0.11	0.86	0.35	0.01	<0.01 *	0.23
9	0	0.94	0.07	0.94	0.83	0.77	0.71	0.39	0.77	0.30	0.06	0.02 *	1.00
6	3	0.44	0.83	0.30	0.77	0.26	0.90	0.06	0.66	0.34	0.04 *	0.13	0.58
6	0	0.55	0.16	0.40	0.92	0.56	0.61	0.27	0.17	0.27	0.11	0.31	0.65
3	0	0.89	0.25	0.81	0.36	0.22	0.20	0.67	0.23	0.66	0.74	0.58	0.21

***Table E2*. Pendelluft analysis.**
Statistical differences among the six studied PEEP levels, at L4.
ANOVA (α=0.05) with Bonferroni's correction for multiple comparisons.
***** to mark statistical differences.
L4 is the layer at 4 cm from the diaphragmatic dome.

ΔVol 0.05 ΔVol 0.10 and ΔVol 0.15 are respectively the delta volumes considered at 0.05, 0.10 and 0.15 seconds.
SB-LowR: spontaneous breathing at low airway resistances;
SB-HIghR: spontaneous breathing at high airway resistances;
MV: mechanical ventilation.

ventilatory modes		Δvol	ROI	PEEP 15	PEEP 12	PEEP 9	PEEP 6	PEEP 3	PEEP 0
L4 - SB-LowR	L4 - SB-HighR	ΔVol0.05	ROI1	0.66	0.87	0.03 *	0.02 *	0.33 *	0.03 *
L4 - SB-LowR	L4 - SB-HighR	ΔVol0.05	ROI2	0.24	0.82	0.22	0.04 *	0.65	0.02 *
L4 - SB-LowR	L4 - SB-HighR	ΔVol0.05	ROI3	0.80	0.88	1.00	1.00	0.62	1.00
L4 - SB-LowR	L4 - SB-HighR	ΔVol0.05	ROI4	0.34	1.00	1.00	1.00	1.00	1.00
L1 - SB-LowR	L1 - SB-HighR	ΔVol0.1	ROI1	0.11	0.23	0.08	0.43	0.61	0.72
L1 - SB-LowR	L1 - SB-HighR	ΔVol0.1	ROI2	0.88	0.51	0.13	0.02 *	0.78	0.76
L1 - SB-LowR	L1 - SB-HighR	ΔVol0.1	ROI3	0.45	0.82	0.01 *	0.04 *	0.96	0.04 *
L1 - SB-LowR	L1 - SB-HighR	ΔVol0.1	ROI4	0.90	0.04 *	0.03 *	0.05 *	0.03 *	0.04 *
L1 - SB-LowR	L1 - SB-HighR	ΔVol0.15	ROI1	0.63	1.00	0.96	0.79	0.62	0.29
L1 - SB-LowR	L1 - SB-HighR	ΔVol0.15	ROI2	0.20	0.52	0.07	0.65	0.99	0.50
L1 - SB-LowR	L1 - SB-HighR	ΔVol0.15	ROI3	0.31	1.00	0.03 *	0.77	0.62	0.03 *
L1 - SB-LowR	L1 - SB-HighR	ΔVol0.15	ROI4	0.69	0.50	0.03 *	0.02 *	0.04 *	0.03 *

***Table E3*. Pendelluft analysis.**
Statistical differences among different vetilatory modes.
ANOVA (α=0.05) with Bonferroni's correction for multiple comparisons. ***** to mark statistical differences.

In the table are reported the statistic that are more descriptive for the findings. To further investigate the effects of resistance on the redistribution of air from non-dependent to dependent region and along a craniocaudal axis, we compared:
SB-LowR (spontaneous breathing at low airway resistances) and SB-HighR (spontaneous breathing at high airway resistances):

1. at cranial level (L4) and in the first time frame (ΔV0.05);
2. At the caudal level (L1) and in the second and third time frame (ΔV0.1 and ΔV0.15).

L1 is the layer at 1 cm from the diaphragmatic dome. L4 is the layer at 4 cm from the diaphragmatic dome. ΔV0.05, ΔV0.1 and ΔV0.15 are respectively the delta volumes considered at 0.05, 0.1 and 0.15 seconds.

PEEP levels		L4 - SB LowR	L1 - SB LowR	L4 - SB HighR	L1 -SB HighR	L4 - MV	L1 - MV
15	12	0.01 *	0.51	0.01 *	0.86	0.01 *	<0.01 *
15	9	0.76	0.73	0.01 *	0.32	0.04 *	<0.01 *
15	6	0.04 *	0.38	0.01 *	0.06	<0.01 *	<0.01 *
15	3	<0.01 *	0.64	<0.01 *	0.13	<0.01 *	<0.01 *
15	0	<0.01 *	0.01 *	<0.01 *	<0.01 *	<0.01 *	<0.01 *
12	9	0.58	0.77	0.38	0.23	0.07	0.03 *
12	6	0.04 *	0.16	0.58	0.06	<0.01 *	<0.01 *
12	3	<0.01 *	0.20	0.29	0.11	<0.01 *	<0.01 *
12	0	<0.01 *	0.00 *	0.10	<0.01 *	<0.01 *	<0.01 *
9	6	0.03 *	0.06	0.72	0.08	<0.01 *	0.08
9	3	<0.01 *	0.43	0.84	0.26	<0.01 *	<0.01 *
9	0	<0.01 *	<0.01 *	0.30	0.01 *	<0.01 *	<0.01 *
6	3	0.01 *	0.59	0.47	0.84	0.34	0.06
6	0	<0.01 *	0.03 *	0.14	0.11	0.05 *	0.04 *
3	0	0.02 *	<0.01 *	0.31	0.03 *	0.11	0.71

***Table E4*. Quadtree decomposition analysis.**
Statistical differences among the six studied PEEP levels.
ANOVA (α=0.05) with Bonferroni's correction for multiple comparisons.***** to mark statistical differences.

L1 (the layer at 1 cm) and L4 (the layer at 4 cm from the diaphragmatic dome).
SB LowR (spontaneous breathing at low airway resistance)
SB HighR (spontaneous breathing at high airway resistance)
MV (mechanical ventilation).

		PEEP 15	PEEP 12	PEEP 9	PEEP 6	PEEP 3	PEEP 0
L1 - SB LowR	L1 - SB HighR	<0.01 *	<0.01 *	<0.01 *	<0.01 *	0.04 *	0.27
L4 - SB LowR	L4 - SB HighR	0.11	0.04 *	0.01 *	0.01 *	0.04 *	0.02 *
L1 - SB LowR	L1 - MV	0.01 *	<0.01 *	<0.01 *	<0.01 *	<0.01 *	<0.01 *
L4 - SB LowR	L4 - MV	0.02 *	<0.01 *	<0.01 *	<0.01 *	<0.01 *	<0.01 *
L1 -SB HighR	L1 - MV	0.86	0.85	0.08	0.03 *	0.01 *	0.03 *
L4 - SB HighR	L4 - MV	0.30	<0.01 *	<0.01 *	<0.01 *	<0.01 *	0.01 *

***Table E5*. Quadtree decomposition analysis.**
Statistical differences among the three studied ventilatory modes:
SB LowR (spontaneous breathing at low airway resistance)
SB HighR (spontaneous breathing at high airway resistance)
MV (mechanical ventilation).

ANOVA (α=0.05) with Bonferroni's correction for multiple comparisons.***** to mark statistical differences

L1 (the layer at 1 cm) and L4 (the layer at 4 cm from the diaphragmatic dome).

L1-L4	PEEP 15	PEEP 12	PEEP 9	PEEP 6	PEEP 3	PEEP 0
SB LowR	0.84	0.25	0.76	0.10	<0.01 *	0.02 *
SB HighR	0.18	0.02 *	0.01 *	0.38	0.18	0.53
MV	0.90	0.54	0.01 *	0.31	0.02 *	0.48

***Table E6*. Quadtree decomposition analysis.**
Statistical differences between the two studied distances from the diaprahgmeatic dome:
L1 (the layer at 1 cm) and
L4 (the layer at 4 cm from the diaphragmatic dome).

ANOVA (α=0.05) with Bonferroni's correction for multiple comparisons.
***** to mark statistical differences between the marked PEEP level and the lower ones.

SB LowR (spontaneous breathing at low airway resistance)
SB HighR (spontaneous breathing at high airway resistance)
MV (mechanical ventilation).
